# Oscillometric, greyscale- and novel color-Doppler-ultrasound indices of macrovascular damage in Sjögren’s: the SICARD cohort study

**DOI:** 10.1186/s13075-025-03625-5

**Published:** 2025-08-01

**Authors:** Konstantinos Triantafyllias, Mirjam Bach, Sebastian Bögel, Muthuraman Muthuraman, George Bertsias, Dimitrios Boumpas, Raoul Bergner, Markus Schepers, Andreas Schwarting

**Affiliations:** 1 Department of Rheumatology, Rheumatology Center, Bad Kreuznach, Germany; 2 Department of Internal Medicine I, Division of Rheumatology and Clinical Immunology, Johannes Gutenberg University Medical Center, Mainz, Germany; 3 Department of Neurology, University Hospital Würzburg, Würzburg, Germany; 4 Department of Rheumatology, Clinical Immunology and Allergy, University Hospital of Heraklion, Heraklion, Greece; 5 Department of Rheumatology and Clinical Immunology, Attikon University Hospital, National and Kapodistrian University of Athens, Athens, Greece; 6 Laboratory of Autoimmunity and Inflammation, Biomedical Research Foundation of the Academy of Athens, Athens, Greece; 7 Department of Rheumatology, Ludwigshafen Hospital, Ludwigshafen, Germany; 8 Institute of Medical Biostatistics, Epidemiology and Informatics, University Medical Center, Johannes-Gutenberg-University, Mainz, Germany

**Keywords:** Sjögren’s, Angiopathy, Aortic stiffness, Carotid atherosclerosis, Cardiovascular risk, SCORE

## Abstract

**Background:**

To assess for the first time a combination of oscillometric, greyscale- and novel color-Doppler ultrasound (US) indices of carotid and aortic damage in patients with primary Sjögren’s syndrome (pSS). Moreover, to examine associations of these markers with patient and disease-characteristics, as well as with a traditional cardiovascular (CV) risk score (SCORE) and its EULAR-modified version (mSCORE).

**Methods:**

Greyscale and color-Doppler indices [resistance (RI)- and pulsatility (PI)-index], as well as markers of atherosclerosis [Intima-Media-Thickness (cIMT), plaques, and cumulative calcification surface], were examined in the common- (CCA) and internal- (ICA) carotid arteries of pSS patients and healthy controls. The gold standard oscillometric marker of aortic stiffness (carotid-femoral pulse wave velocity; cfPWV) and the traditional SCORE/mSCORE, were also assessed.

**Results:**

We recruited *119* pSS-patients and *97* controls. Patients exhibited significantly higher cfPWV (*p*_*adj*_ = *0.025*), cIMT (*p*_*adj*_ < *0.001*), and calcification area (*p* = *0.013)*, compared to controls. According to mSCORE, *5.7%* of the patients had high CV risk. However, cfPWV and carotid-sonography revealed increased aortic stiffness in *45.4%* and carotid atherosclerosis in *69.2%*, respectively. Among pSS-patients, cfPWV correlated with C-reactive-protein (*rho* = *0.325, p* < *0.001*), erythrocyte-sedimentation-rate (*rho* = *0.271*, *p* = *0.003*), and traditional CV-risk factors (age, cholesterol, systolic blood pressure: all; *p* < *0.01*). ICA-RI and ICA-PI were higher in patients with further (non-rheumatological) autoimmune diseases (both; *p* < *0.05*).

**Conclusion:**

In the largest cfPWV/US-cohort examined to date, pSS-patients had significantly higher aortic stiffness and atherosclerosis than controls. Aortic stiffness was predicted by systemic inflammation, alongside traditional CV risk factors. cfPWV and carotid-US may help identify subclinical end-organ disease and atherosclerosis and thus assist CV/CVB-screening in pSS.

**Trial registration:**

DRKS00031470.

**Supplementary information:**

The online version contains supplementary material available at 10.1186/s13075-025-03625-5.

## Background

Primary Sjogren´s Syndrome (pSS) is an autoimmune disease characterized by inflammation of the exocrine glands, leading to functional impairment [1, 2]. Up to 70% of patients suffer from an additional systemic organ involvement involving the lungs, kidneys, central and peripheral nervous system, as well as the gastrointestinal tract, and cutaneous tissue [3, 4]. Moreover, pSS patients are under increased cardiovascular (CV) risk [2, 5, 6] with higher rates of CV involvement compared to the general population (61.6% vs. 29.7%) [7].

In pSS patients, an increased rate of cerebrovascular (CVB) events (2.5% vs. 1.4%) and myocardial infarction (1.0% vs. 0.4%), compared to healthy controls has been described [8]. In addition, a meta-analysis evaluating 14 studies showed a higher relative risk for coronary- (1.34), CVB- (1.46), and thromboembolic- morbidity (1.78) [9] and accordingly, a need for early CVR detection and prevention has been highlighted [7].

The 2022 EULAR recommendations for CV risk management in rheumatic diseases (including pSS) emphasized the importance of screening and controlling CV risk factors in rheumatic diseases, including pSS. The use of traditional CV prediction tools has been suggested for patients with pSS [10]. One of these tools is SCORE (Systematic Coronary Risk Evaluation), which is widely used for CV risk classification in the general population and has been previously recommended by EULAR for CV risk assessment in patients with inflammatory arthropathies [11, 12]. However, SCORE considers only traditional CV risk factors, such as nicotine use, lipids, systolic blood pressure, age, and gender. Thus, the effects of systemic inflammation and further disease-associated factors (i.e. medication, impaired physical fitness) are not taken into account [11, 13]. For this reason, the EULAR guidelines for CV risk management recommended the adaptation of such CV risk prediction models by a 1.5 multiplication factor in the case of rheumatoid arthritis (RA) and other inflammatory arthritides [14]. Nevertheless, no conclusive evidence regarding precise means of CV risk calculation is available in pSS and the applicability of the SCORE in patients with pSS has not been adequately examined. For this reason, there is a need for additional biomarkers that can help us achieve a more accurate assessment of true CV risk [15, 16].

Stiffness of the large arteries is one of the best-validated CV surrogates in the general population, showing a high positive predictive value for future CV events [17]. In particular, stiffness of the aortic vasculature, measured by the oscillometric method of carotid-femoral pulse wave velocity (cfWV), has been shown to predict CV events accurately and has been suggested for primary CV risk prevention in the general population [17]. For instance, a thorough meta-analysis, comprising 17 studies and a total of 15,877 subjects, revealed that individuals with elevated aortic pulse wave velocity (PWV) values had significantly higher pooled relative risks (RR) for CV mortality compared to those with low aortic PWV (RR: 2.02, 95% CI: 1.68 to 2.42) [18]. Moreover, carotid sonography is a well-established atherosclerosis assessment tool. Its value in risk stratification and concomitant plaque identification has been repeatedly shown both in the general population and in patients with rheumatic diseases [18, 19]. During the last years, our research group and others have examined cfPWV and carotid sonography in the context of various autoimmune diseases, such as rheumatoid arthritis (RA) [20], psoriatic arthritis [21], mixed connective tissue disease [22], systemic lupus erythematosus (SLE) [23], antisynthetase syndrome [16] and fibromyalgia [24].

Until recently, greyscale sonography markers [carotid Intima-Media-Thickness (cIMT), plaque formation] have been mostly examined in patients with pSS. Carotid Doppler indices, like Resistance- (RI) and Pulsatility- index (PI) have only been sparsely evaluated in two small pSS case series with *n* = 22 and *n* = 18 patients respectively [25, 26]. This constitutes a probable literature gap since these markers can provide information on carotid compliance and thus be indicative of a higher risk for ischemic stroke [27, 28].

A recent metanalysis of existing PWV (*n* = 3) and cIMT (*n* = 5) studies in pSS, revealed overall higher cfPWV- and cIMT-values than controls [5]. However, a more recent study found no differences between a small group of patients (*n* = 30) and controls (*n* = 30) [29]. Generally, available data on CV surrogates in pSS arise from low-numbered, cross-sectional studies with a limited number of macroangiopathy and CV risk markers. Moreover, we are unaware of the associations between these markers and traditional CV risk tools like SCORE and the EULAR mSCORE.

In this study, we aimed to simultaneously assess aortic stiffness (measured by cfPWV) and a combination of novel (PI, RI) and well-established (cIMT, plaques, calcification burden) carotid angiopathy US markers in the largest—to date—pSS cohort examined. These findings were compared with those of healthy controls to evaluate subclinical aortic and carotid stiffness, as well as carotid atherosclerosis in patients with pSS. Moreover, associations of this extended CV surrogate panel with patient and disease-specific characteristics, as well as with the traditional SCORE and its EULAR-modified version (mSCORE) were evaluated.

## Methods

### Study population and design

The prospective’’Sjögren syndrome associated CARDiovascular risk (SICARD)’’ cohort consists of consecutive inpatients with known pSS being treated at the Acute Rheumatology Center Rhineland-Palatinate and the University Medical Center of Mainz in Germany. Hospital co-workers without any inflammatory disease, who freely agreed to participate following an open call, served as a control group. All patients and controls underwent cfPWV examinations. Moreover, subgroups of the two collectives were examined via B-mode and Doppler sonography of the common- (CCA) and internal- (ICA) carotid arteries. This study is part of the multicenter CARD cohorts, which initially focused solely on measuring aortic stiffness via cfPWV. Sonographic examinations of the carotid arteries were incorporated at a later stage, following a modification and expansion of the study design. From that point onward, all patients underwent a comprehensive assessment, including oscillometric cfPWV measurement, grayscale ultrasound (GSUS), and color Doppler ultrasound (CDUS). Excluded from both groups were individuals aged < 18 years, patients with a malignant disease, and patients with a missing capacity to consent. Moreover, patients with current or past cardiovascular, cerebrovascular, or peripheral arterial disease were excluded from the study. All included patients fulfilled the 2016 ACR/EULAR classification criteria (score ≥ 4) [30]. The study has been approved in adherence to the Helsinki Declaration by the ethics committee of Rhineland Palatinate State Medical Council, Germany.

### Data collection

We documented demographic characteristics and the presence of traditional CV risk factors, like arterial hypertension and type 2 diabetes (patient´s history), dyslipidemia during the inpatient stay, and cigarette smoking in both groups. Moreover, we documented the current intake of antihypertensive drugs and statins. Additionally, laboratory examinations were performed including inflammation markers [C-Reactive Protein (CRP), erythrocyte sedimentation rate (ESR)], differential blood counts, renal parameters and autoantibodies [by ELISA: Rheumatoid factor (RF), double-stranded DNA, SSA-Ro, SSB-La, Sm, Scl-70, Jo-1, U1-RNP and by indirect immunofluorescence on HEp-2 cells: antinuclear antibodies (ANA)].

Moreover, we reported systemic involvement as defined by the presence of pulmonary, cutaneous, renal, lymphatic, glandular, or haematologic manifestations in the context of the disease. In particular, pulmonary involvement was screened by chest X-ray in two planes and lung function tests. In case of typical ILD symptoms (i.e. cough or dyspnea) and/or abnormalities in the pulmonary screening examinations, an additional high-resolution computer tomography (HRCT) scan of the lungs was performed. Cutaneous manifestations included known purpura, urticaria, cutaneous vasculitis, erythema multiforme-like lesions, and erythema nodosum. Renal involvement was reported as present in the case of distal renal tubular acidosis or glomerulonephritis. Lymphatic and glandular abnormalities were determined as clinical and/or sonographic diagnosed lymph node enlargement and submandibular and/or parotid gland enlargement, respectively. Hematologic abnormalities were defined as leucopenia, anemia, thrombocytopenia, monoclonal gammopathy, and/or the confirmed diagnosis of a hematologic disease. ESSDAI was calculated according to the EULAR guidelines [31].

We documented the presence of arthralgia, and clinically defined arthritis as the simultaneous occurrence of joint swelling and tenderness. Moreover, we registered the presence of further non-rheumatological autoimmune diseases via patient´s history like Hashimoto’s thyroiditis, Basedow’s disease, inflammatory bowel diseases, vitiligo, primary biliary cholangitis, autoimmune hepatitis and gastritis, multiple sclerosis, as well as myasthenia gravis. The currently taken conventional and biologic disease-modifying anti-rheumatic drugs (csDMARD and bDMARDS, respectively) and the current glucocorticoid therapy were also documented. Furthermore, we registered the subjective experience of sicca symptoms, the Schirmer´s test (abnormal if < 5 mm after 5 min), and Saxon´s test (not part of the classification, abnormal if < 2.5 g after 2 min) [30].

### cfPWV and carotid sonography

As described previously [16, 24], the examination of cfPWV was carried out according to the manufacturer´s instructions of a validated non-invasive oscillometric device (Vicorder®, SMT medical, Wuerzburg, Germany) by trained medical staff and following the expert consensus statement on the measurement of aortic stiffness [32]. The medical staff was aware of case status, but was blinded to other surrogate measures, such as carotid ultrasound results, laboratory values, and the individual’s medical history. cfPWV was calculated by dividing (0.8 x) traveled pulse wave distance (right CCA—right femoral artery) by pulse transit-time (meters/seconds; m/s) and the average value of 3 measurements was documented [33]. cfPWV values > 10 m/s were considered as an indicator of increased CV risk [32].

Carotid greyscale (GSUS) and color Doppler (CDUS) ultrasound was performed by an experienced examiner [K.T., rheumatologist, certified trainer of the German Society of Ultrasound in Medicine (DEGUM)]. For the measurements, a linear transducer of a MyLab9-US device (Esaote®) operating at a frequency of 15 MHz was used. The evaluation of cIMT was performed in both CCA at the end-diastolic phase, approximately 1 cm proximal to the carotid bulb, at three subsequent spots. Only the maximum value was taken into further account. A localized thickening > 1.2 mm in either carotid bulb, was documented as an atherosclerotic plaque [34]. The cumulative calcification area on both sides was measured and documented. Patients and controls with cIMT > 0.9 mm in the CCA and/or ≥ 1 plaque(s) on either side were classified as having subclinical carotid atherosclerosis [16, 34].

CDUS consisted of the bilateral duplex assessments of mid-CCA and proximal ICA in two planes. As soon as there were multiple identical waveforms in the wave flow spectrum for 5 s, they were documented regarding peak systolic (PSV) and end-diastolic velocity (EDV). Subsequently, the software calculated the RI according to the Pourcelot formula [RI = (PSV-EDV)/PSV] [35] and the PI according to the Golsing formula [PSV-EDV)/mean flow velocity (MFV)] [36].

### SCORE/mSCORE calculation

SCORE was calculated according to the guidelines of the European Society of Cardiology (ESC) [11] excluding patients with former CV events. The modified SCORE (mSCORE) was calculated by multiplying SCORE by 1.5 [14]. Since no specific suggestions for pSS have been made, we have also included mSCORE in our statistical assessments. Patients with SCORE/mSCORE > 5% are considered to be at high risk [37].

### Statistical analysis

The normality assumption was evaluated by quantile–quantile plots and the Shapiro–Wilk test. Normally distributed variables were presented as mean (standard deviation), whereas skewed ones as median (25th/75th percentiles). Categorical variables were summarized as absolute (n) and relative (%) frequencies. The chi-squared test was used for their comparison. Comparisons of cfPWV, cIMT, PI, and RI between pSS patients and controls were done through a t-test or the Mann–Whitney-U test as appropriate. The same applies to the evaluation of the association between the mentioned markers and categorial variables with two categories. The examination of the correlations between cfPWV, cIMT, PI, RI, and continuous characteristics was performed by Spearman´s (rho) and Pearson´s (r) correlation coefficients. These statistical calculations were performed by using IBM SPSS® 23 V5 software (USA).

Additionally, we examined the difference of cfPWV, cIMT, PI, and RI between pSS patients and the control group after controlling for possible confounding factors by multiple regression logistic analyses.

A propensity score analysis was performed in order to address potential confounding (in a way that separates design and analysis) and to get causal inference estimates. The propensity score was calculated via a logistic regression (of treated vs untreated) in terms of age, sex, body mass index (BMI), diabetes mellitus, mean arterial pressure (MAP) and smoking (these variables were selected based on clinical practice and expertise). The propensity score was estimated using a complete case analysis. Later the propensity score was used to get a 1:1-matching (by sampling without replacement), using the matching method “genetic”, with caliper (for propensity score) set to 0.1, of R’s matchit function (from the MatchIt package). Balance was checked using the standardized mean difference and diagnostic plots. The matched samples were analyzed with paired t-tests.

These statistical calculations were performed by using the software R (version 4.2.2).

A probability value below 0.05 was considered statistically significant.

## Results

We performed cfPWV measurements in 119 pSS patients and 97 healthy controls. Additionally, a subgroup of 52 patients and 46 controls underwent carotid GSUS and CDUS examinations. Eligible patients and controls for the calculation of SCORE were subjects with an age between 40 and 70 years old (88 pSS patients and 63 controls). Tables 1 and 2 show the descriptive characteristics and statistical differences of patients with pSS and the control group. Tables S.1. and S.4. show additional patient characteristics separately (Supplementary Material).

**Table 1:** Descriptive characteristics by group (controls vs. cfPWV patient group)

	Controls (*n* = 97)	Patients (*n* = 119)	Significance (P)
Age† *(years)*	48.25 ± 12.96	55.29 ± 13.37	< 0.001***
Gender *(female)*	81 (83.5%)	111 (93.3%)	0.023*
BMI *(kg/m*^*2*^*)*	24.73 ± 4.20	27.38 ± 5.90	< 0.001***
Arterial hypertension *(yes)*	19 (20.0%)	47 (39.5%)	0.002**
Diabetes Mellitus 2 *(yes)*	1 (1.1%)	5 (4.2%)	0.166
Nicotine *(smokers)*	15 (15.8%)	16 (13.4%)	0.628
HDL† *(mg/dl)*	67.13 ± 18.15	66.38 ± 17.28	0.788
LDL† *(mg/dl)*	128.94 ± 37.56	131.35 ± 47.40	0.717
Total Cholesterol† *(mg/dl)*	211.08 ± 43.97	199.78 ± 44.97	0.074
Blood pressure systolic† *(mmHg)*	123.13 ± 15.50	119.51 ± 16.54	0.110
Blood pressure diastolic† *(mmHg)*	78.13 ± 10.48	75.58 ± 9.73	0.073
MAP† *(mmHg)*	93.14 ± 11.42	90.19 ± 10.74	0.060
Antihypertensive therapy *(yes)*	19 (20.2%)	39 (36.1%)	0.013*
*Monotherapy*		*18 (46.2%)*	
*Dual therapy*		*10 (25.6%)*	
*Triple or more*		*11 (28.2%)*	
*Sartans*		*14 (35.9%)*	
*ACE-Inhibitors*		*15 (38.5%)*	
*Diuretics*		*10 (25.6%)*	
*Beta-Blockers*		*11 (28.2%)*	
*Calcium Channel Blockers*		*17 (43.6%)*	
Statine therapy *(yes)*	4 (4.1%)	13 (10.9%)	0.065
Heart rate ‡ *(/min)*	66 (59–70)	74 (66.59–81.08)	< 0.001***
cfPWV † *(m/s)*	8.62 ± 1.57	10.01 ± 2.26	< 0.001***
SCORE ‡ *(%)*	1 (0–2)	1 (1–2)	0.392
mSCORE ‡ *(%)*	1.5 (0–3)	1.5 (1.5–3)	0.392

^‡^ Non-normal distribution: presentation as median (interquartile range)

^†^ Normal distribution: presentation as mean (S.D.). Others: absolute and relative frequencies

^*^^−^ *** Significant difference between the two groups

*HDL* high-density lipoprotein, *LDL* low-density lipoprotein, *SCORE* Systemic COronary Risk Evaluation

**Table 2:** Descriptive characteristics by group (carotid sonography subgroups)

	Controls (*n* = 46)	Patients (*n* = 52)	Significance (P)
Age† *(years)*	52.57 ± 10.34	54.52 ± 12.78	0.411
Gender *(female)*	35 (76.1%)	49 (94.2%)	0.018*
BMI † *(kg/m*^*2*^*)*	26.24 ± 4.20	26.56 ± 5.52	0.750
Arterial hypertension *(yes)*	14 (30.4%)	16 (30.8%)	1
Diabetes Mellitus 2 *(yes)*	1 (2.2%)	4 (7.7%)	0.371
Nicotine *(smokers)*	8 (17.4%)	9 (17.3%)	1
HDL† *(mg/dl)*	65.08 ± 15.23	67.87 ± 17.15	0.488
LDL† *(mg/dl)*	136.11 ± 36.37	133.47 ± 39.15	0.777
Total Cholesterol† *(mg/dl)*	212.69 ± 43.59	202.52 ± 39.33	0.257
Blood pressure systolic† *(mmHg)*	125.53 ± 16.22	117.40 ± 15.67	0.019*
Blood pressure diastolic† *(mmHg)*	80.26 ± 10.52	75.25 ± 9.57	0.022*
MAP † *(mmHg)*	95.35 ± 11.78	89.48 ± 10.41	0.015*
Blood pressure therapy *(yes)*	12 (26.1%)	14 (26.9%)	1
Statins	4 (8.7%)	7 (13.5%)	0.505
cIMT *(mm)* †	0.77 ± 0.13	0.93 ± 0.18	< 0.001***
Calcification area *(cm*^*2*^*)* ‡	8 (0–17)	17 (7–28.7)	0.013*
CCA-PI ‡	1.54 (1.32–1.84)	1.64 (1.37–1.88)	0.168
CCA-RI †	0.72 ± 0.09	0.76 ± 0.12	0.133
ICA-PI †	1.08 ± 0.29	1.11 ± 0.31	0.648
ICA-RI ‡	0.60 (0.54–0.68)	0.62 (0.54–0.69)	0.674

^‡^ Non-normal distribution: presentation as median (interquartile range)

^†^ Normal distribution: presentation as mean (S.D.). Others: absolute and relative frequencies

^*^^−^ *** Significant difference between the two groups

*HDL* high-density lipoprotein, *LDL* low-density lipoprotein, *ENA* extractable nuclear antigens, *ESR* erythrocyte sedimentation rate, *CRP* C-reactive protein, *ANA* antinuclear antibodies, *ESSDAI* EULAR Sjögren's syndrome disease activity index, *cIMT* carotid intima-media thickness, *CCA* common carotid arteries, *ICA* internal carotid artery, *RI* resistive index, *PI* pulsatility index

### Association between group status (pSS vs control): cfPWV, Carotid sonography, and SCORE

**Fig. 1:**
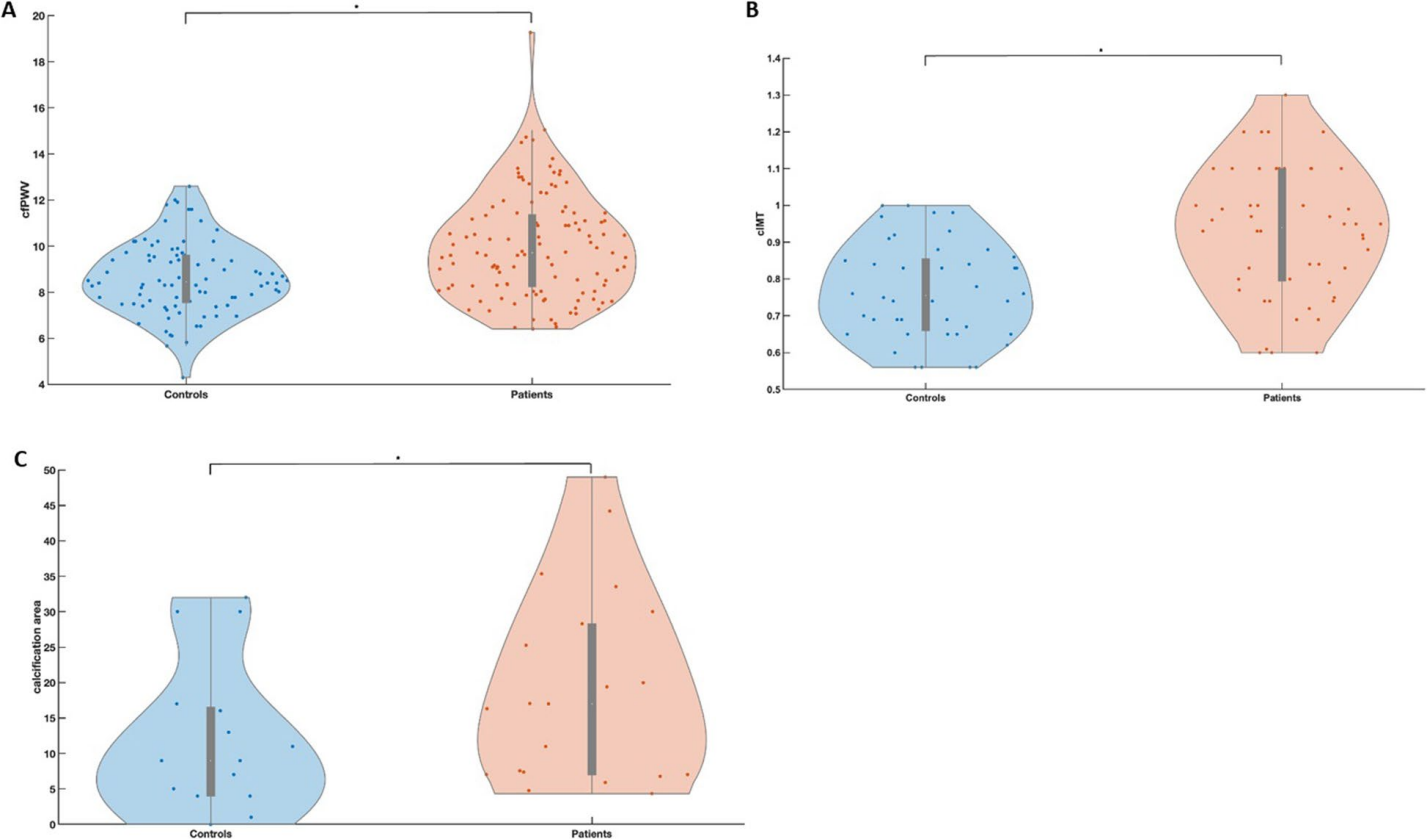
Distribution of values in control subjects and pSS patients. **A** cfPWV; **B** cIMT; **C** calcification area. All *p* < 0.05*. pSS: primary Sjogren´s Syndrome, cfPWV: carotid-femoral pulse wave velocity, cIMT: carotid intima media thickness

Mean cfPWV was significantly higher in patients with pSS compared with control subjects [10.01 ± 2.26 vs. 8.62 ± 1.57; *p* < 0.001] (Table 1, Fig. 1).

cfPWV remained statistically significantly higher in the patient group even after regression adjustment analysis for the effects of possible confounding factors and parameters which were different in the two groups like age, gender, BMI, arterial hypertension, hyperlipidemia, and smoking status (1.329, 95%CI: 1.043–1.852; p_adj_ = 0.025) (Supplementary Material Table S.2.).

This was also the case when we adjusted exclusively for factors that were different in the whole patient cohort and in the control group (age, gender, BMI, arterial hypertension, antihypertensive therapy and heart rate; 0.687, 95%CI: 0.003–1.370; *p* = 0.049) (Supplementary Material Table S.3.).

cIMT and the calcification area (cumulated surface of atherosclerotic plaques) were also significantly higher in the subgroup of pSS patients, compared to the subgroup of control subjects [0.93 ± 0.18 vs. 0.77 ± 0.13; *p* < 0.001 and 17 (7–28.7, IQR) vs 8 (0–17, IQR); *p* = 0.013] (Table 2, Fig. 1).

Regression analysis accounting for the effects of possible confounding factors, as well as parameters that were different in the two groups like age, gender, hyperlipidemia, arterial hypertension, smoking status and diabetes showed that cIMT was significantly higher in the patient group compared to the control group even after statistical adjustment (2.329, 95%CI: 3.03–34.81; p_adj_ < 0.001) (Supplementary Material Table S.5.).

This was also the case when we adjusted exclusively for factors that were different in the whole patient cohort and in the control group (gender, mean, diastolic and systolic arterial pressure; 0.134, 95%CI: 0.053–0.215; *p* = 0.002) (Supplementary Material Table S.6.).

As for the carotid Doppler indices (RI and PI), there was no significant difference between the patient and control subgroups (Table 2). The same occurred for SCORE, which was not significantly higher in patients compared with controls [1 (1–2, IQR) vs 1 (0–2, IQR); *p* = 0.392] (Table 1).

### Propensity score matching

Propensity score matching was performed to create a 1:1 matched sample of pSS patient and control subgroups, based on age, sex, BMI, diabetes mellitus, MAP, and smoking status. This matching resulted in 58 matched pairs (116 subjects in total), from an initial pool of 202 participants (pSS patients and controls). The matched analysis confirmed a statistically significant difference in carotid-femoral pulse wave velocity (cfPWV) between the pSS patients and controls, with an average treatment effect estimate of 0.718 (standard error: 0.323, *p* = 0.028).

Propensity score matching was also performed within the carotid ultrasound subgroup based on the same parameters (age, sex, BMI, diabetes mellitus, MAP, and smoking status), resulting in 27 matched pairs (1:1; 54 subjects in total), from an initial pool of 86 participants (pSS patients and controls). This analysis confirmed the previously observed difference in carotid intima-media thickness (cIMT) between pSS patients and controls, with an estimated average treatment effect of 0.147 (standard error: 0.046, *p* = 0.002). However, no statistically significant differences were found between the groups in terms of the Doppler indices ACC-PI, ACC-RI, ACI-RI, or ACI-PI.

### Associations of cfPWV with group and disease characteristics

**Fig. 2:**
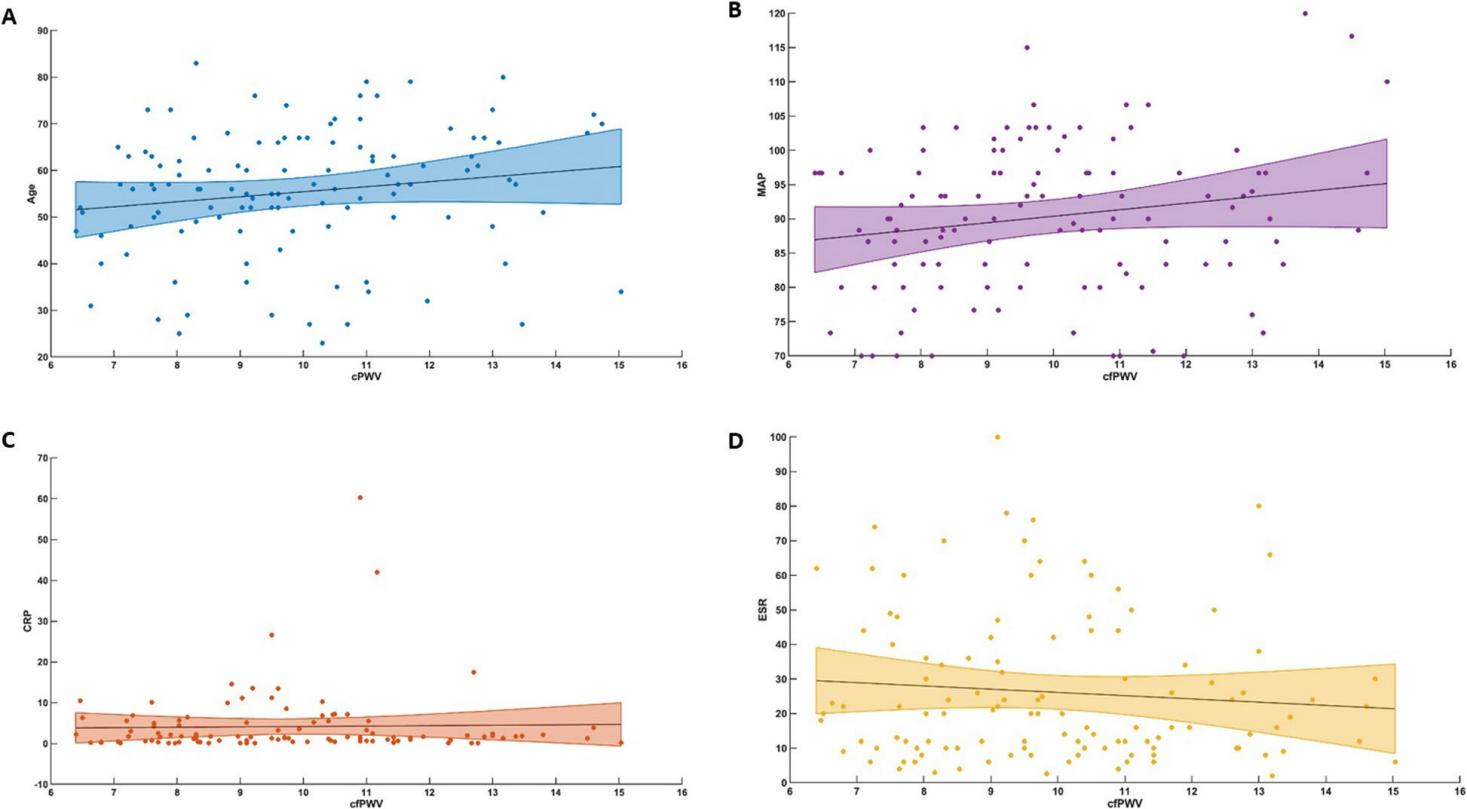
Associations between cfPWV and traditional CV-factors and inflammation markers in patients with pSS. **A** Age; **B** MAP; **C** CRP; **D** ESR; *all; p* < *0.05*. *cfPWV:* carotid-femoral pulse wave velocity*, CV:* cardiovascular, *MAP:* mean arterial pressure, *CRP*: c-reactive protein, *ESR*: erythrocyte sedimentation rate

Within the patient group, unadjusted analyses revealed an association of cfPWV with age (*r* = 0.631, *p* < 0.001), systolic blood pressure (*r* = 0.479, *p* < 0.001), total cholesterol (*r* = 0.253, *p* = 0.006), ESR (rho = 0.271, *p* = 0.003) and CRP (rho = 0.325, *p* < 0.001) (Fig. 2).

Additionally, higher cfPWV values could be found in patients with arterial hypertension compared to patients without [11.00 ± 1.4 m/s vs. 9.37 ± 2.36 m/s; *p* < 0.001]. The same could be seen for diabetes [12.15 ± 2.58 m/s vs. 9.91 ± 2.21 m/s; *p* = 0.029] and hyperlipidemia [10.46 ± 2.41 m/s vs. 9.58 ± 2.03 m/s; *p* = 0.034], respectively (Table 3).

**Table 3:** Associations between cfPWV, cIMT, and patient characteristics

	cfPWV †		cIMT †	
	*rho/r*	*P*	*rho/r*	*P*
Age† *(years)*	0.631	< 0.001***	0.634	< 0.001***
HDL† *(mg/dl)*	0.032	0.785	0.073	0.702
LDL† *(mg/dl)*	0.223	0.054	−0.112	0.555
Total Cholesterol† *(mg/dl)*	0.253	0.006**	0.126	0.375
Blood pressure systolic† *(mmHg)*	0.479	< 0.001***	0.323	0.020*
Blood pressure diastolic† *(mmHg)*	0.176	0.060	0.094	0.511
Rheumatoid factor‡	0.093	0.337	−0.006	0.969
ESR‡ *(mm/h)*	0.271	0.003**	0.205	0.145
CRP‡ *(mg/l)*	0.325	< 0.001***	0.093	0.514
ANA ‡ *(titer)*	−0.005	0.961	0.083	0.589
Saxon´s test difference‡ *(g)*	0.013	0.916	−0.267	0.187
Schirmer´s test (lowest value *(mm)*) ‡	0.006	0.959	0.251	0.226
mSCORE ‡ *(%)*	0.514	< 0.001***	0.43	0.005**
ESSDAI ‡	0.123	0.393	0.234	0.320
	*Mean (S.D.)*	*P*	*Mean (S.D.)*	*P*
Gender				
*Female*	10.03 (2.27)	0.751	0.92 (0.17)	0.100
*Male*	9.76 (2.14)		1.09 (0.12)	
Arterial hypertension				
*Yes*	11.00 (1.4)	< 0.001***	1.01 (0.16)	0.02*
*No*	9.37 (2.36)		0.89 (0.17)	
Diabetes Mellitus 2				
*Yes*	12.15 (2.58)	0.029*	1.04 (0.13)	0.165
*No*	9.91 (2.21)		0.92 (0.18)	
Hyperlipidemia				
*Yes*	10.46 (2.41)	0.034*	0.95 (0.17)	0.258
*No*	9.58 (2.03)		0.90 (0.18)	
Nicotine				
*Yes*	9.63 (1.90)	0.467	0.90 (0.13)	0.602
*No/Ex*	10.07 (2.31)		0.93 (0.19)	
ENA-Screen				
*Positive*	9.92 (2.29)	0.752	0.92 (0.16)	0.861
*Negative*	10.06 (2.25)		0.93 (0.21)	
SSA-Antibodies				
*Positive*	9.72 (2.03)	0.358	0.92 (0.17)	0.972
*Negative*	10.10 (2.22)		0.92 (0.21)	
SSB-Antibodies				
*Positive*	9.04 (1.55)	0.073	0.89 (0.19)	0.455
*Negative*	10.00 (2.16)		0.93 (0.18)	
Hypergammaglobulinaemia				
*Positive*	10.06 (2.80)	0.980	0.97 (0.18)	0.439
*Negative*	10.07 (2.03)		0.90 (0.13)	
Saxon´s test				
*Positive*	9.55 (2.44)	0.271	0.93 (0.19)	0.767
*Negative*	10.19 (1.96)		0.95 (0.19)	
Schirmer´s test				
*Positive*	9.73 (2.16)	1	0.90 (0.19)	0.141
*Negative*	9.84 (1.95)		1.07 (0.20)	
Sicca symptoms				
*Yes*	10.08 (2.31)	0.299	0.93 (0.18)	0.647
*No*	9.21 (1.69)		0.98 (0.11)	
Systemic involvement				
*Yes*	10.08 (2.31)	0.706	0.93 (0.18)	0.560
*No*	9.21 (1.69)		0.98 (0.11)	
Hematological involvement ‡				
*Lymph node swelling*	10.13 (1.88)		0.90 (0.17)	
*Hem. disease*	9.93 (1.70)	0.989	0.97 (0.33)	0.814
*No*	10.06 (2.35)		0.94 (0.18)	
Parotid enlargement				
*Yes*	9.32 (1.90)	0.319	0.92 (0.15)	0.880
*No*	10.11 (2.31)		0.93 (0.18)	
Glucocorticoid therapy				
*Yes*	10.24 (2.60)	0.453	0.91 (0.14)	0.665
*No*	9.91 (2.05)		0.94 (0.19)	
Disease-modifying antirheumatic drugs				
*Yes*	9.90 (2.21)	0.494	0.93 (0.16)	0.991
*No*	10.19 (2.29)		0.93 (0.20)	
Further autoimmune diseases				
*Yes*	9.69 (1.87)	0.472	0.90 (0.16)	0.608
*No*	10.08 (2.34)		0.93 (0.18)	

^‡^ Non-normal distribution

^†^ Normal distribution. Quantitative characteristics: Spearmann’s test (non-normal distribution; rho), Pearson´s test (normal distribution; r). Qualitative characteristics: Mann–Whitney-U-test, ANOVA

^*^^−^ *** Significant difference between the two groups

*HDL* high-density lipoprotein, *LDL* low-density lipoprotein, *ENA* extractable nuclear antigens, *ESR* erythrocyte sedimentation rate, *CRP* C-reactive protein, *ANA* antinuclear antibodies, *SCORE* Systemic COronary Risk Evaluation, *ESSDAI* EULAR Sjögren's syndrome disease activity index, *cfPWV* Carotid-femoral pulse-wave velocity, *cIMT* carotid intima-media thickness

There was no significant association between cfPWV and ESSDAI (*p* > 0.05).

Among the control group, we were able to show correlations of cfPWV with age (*r* = 0.69, *p* < 0.001), total cholesterol (rho = 0.25, *p* = 0.007), BMI (*r* = 0.22, *p* = 0.02) and MAP (*r* = 0.325, *p* < 0.001). Moreover, subjects with arterial hypertension had higher cfPWV values in comparison to subjects without [10.59 ± 1.85 m/s vs 8.89 ± 2.10 m/s; *p* = 0.049].

### Associations of carotid sonography parameters with group and disease characteristics

Tables 3 and 4 depict the results of the correlation analyses between cIMT, CCA (RI, PI), ICA (RI, PI), and patient characteristics.

**Table 4:** Associations between CCA (RI, PI) and ICA (RI, PI) and patient characteristics

	CCA				ICA			
	RI †		PI ‡		RI ‡		PI †	
	*rho/r*	*P*	*rho/r*	*P*	*rho/r*	*P*	*rho/r*	*P*
Age† *(years)*	0.097	0.520	0.117	0.446	0.481	0.002 **	0.457	0.003 **
HDL† *(mg/dl)*	−0.207	0.301	−0.063	0.760	0.002	0.992	−0.091	0.689
LDL† *(mg/dl)*	−0.385	0.047*	−0.340	0.090	−0.228	0.307	−0.252	0.258
Total Cholesterol† *(mg/dl)*	−0.252	0.092	−0.196	0.198	−0.103	0.529	−0.093	0.570
Blood pressure systolic† *(mmHg)*	0.251	0.092	0.203	0.180	0.465	0.002 **	0.442	0.004**
Blood pressure diastolic† *(mmHg)*	0.188	0.217	0.196	0.203	0.210	0.198	0.217	0.185
Rheumatoid factor ‡	−0.50	0.748	−0.170	0.280	−0.113	0.499	−0.204	0.220
ESR *(mm/h)*‡	0.245	0.101	0.151	0.322	−0.040	0.805	−0.021	0.897
CRP *(mg/l)*‡	0.152	0.313	0.017	0.913	0.112	0.492	0.071	0.662
ANA *(titer)*‡	0.232	0.150	0.121	0.463	−0.031	0.858	−0.086	0.624
Saxon´s test difference‡ *(g)*	0.014	0.950	0.189	0.388	0.052	0.827	0.106	0.657
Schirmer´s test (lowest value) ‡ *(mm)*	0.178	0.405	0.189	0.376	−0.094	0.676	−0.145	0.520
mSCORE ‡ *(%)*	0.139	0.404	0.035	0.839	0.420	0.015*	0.354	0.044*
	*Mean S.D*	*P*	*Mean S.D*	*P*	*Mean S.D*	*P*	*Mean S.D*	*P*
Gender								
*Female*	0.75 (0.10)	0.095	1.71 (0.48)	0.210	0.63 (0.11)	0.805	1.11 (0.31)	0.777
*Male*	0.87 (0.28)		2.11 (1.12)		0.60 (-)		1.02 (-)	
Arterial hypertension								
*Yes*	0.84 (0.19)	0.003**	2.18 (0.30)	0.001**	0.68 (0.07)	0.080	1.27 (0.27)	0.049*
*No*	0.73 (0.06)		1.59 (0.30)		0.61 (0.11)		1.05 (0.31)	
Diabetes Mellitus 2								
*Yes*	0.78 (0.06)	0.669	1.66 (0.38)	0.778	0.67 (0.02)	0.491	1.26 (0.06)	0.329
*No*	0.75 (0.12)		1.74 (0.55)		0.62 (0.11)		1.10 (0.32)	
Hyperlipidemia								
*Yes*	0.72 (0.07)	0.075	1.59 (0.31)	0.071	0.62 (0.12)	0.891	1.10 (0.31)	0.818
*No*	0.79 (0.15)		1.87 (0.66)		0.63 (0.09)		1.12 (0.31)	
Nicotine								
Smokers	0.75 (0.18)	0.944	1.65 (0.75)	0.632	0.59 (0.11)	0.303	0.98 (0.28)	0.242
Non/Ex-Smokers	0.76 (0.11)		1.75 (0.48)		0.63 (0.10)		1.14 (0.31)	
ENA-Screen								
*Positive*	0.75 (0.11)	0.576	1.67 (0.52)	0.314	0.63 (0.11)	0.491	1.11 (0.32)	0.852
*Negative*	0.77 (0.14)		1.85 (0.57)		0.61 (0.11)		1.09 (0.33)	
SSA-Antibodies								
P*ositive*	0.75 (0.12)	0.536	1.67 (0.53)	0.289	0.62 (0.08)	0.655	1.08 (0.28)	0.539
*Negative*	0.77 (0.14)		1.86 (0.55)		0.63 (0.15)		1.15 (0.38)	
SSB-Antibodies								
*Positive*	0.70 (0.06)	0.130	1.44 (0.24)	0.040*	0.59 (0.06)	0.276	0.95 (0.15)	0.083
*Negative*	0.77 (0.13)		1.83 (0.59)		0.64 (0.12)		1.15 (0.35)	
Hypergammaglobulinemia								
*Positive*	0.81 (0.20)	0.404	1.98 (0.92)	0.442	0.61 (0.10)	0.559	1.08 (0.38)	0.569
*Negative*	0.76 (0.11)		1.77 (0.47)		0.65 (0.12)		1.17 (0.34)	
Saxon´s test								
*Positive*	0.74 (0.79)	0.399	1.69 (0.59)	0.513	0.64 (0.07)	0.760	1.14 (0.28)	0.817
*Negative*	0.78 (0.14)		1.86 (0.58)		0.65 (0.16)		1.17 (0.39)	
Schirmer´s test								
*Positive*	0.75 (0.12)	0.968	1.72 (0.58)	0.914	0.64 (0.11)	0.938	1.14 (0.30)	0.889
*Negative*	0.75 (0.06)		1.76 (0.49)		0.65 (0.12)		1.11 (0.38)	
Sicca symptoms								
*Yes*	0.75 (0.12)	0.751	1.72 (0.54)	0.601	0.62 (0.11)	0.311	1.10 (0.31)	0.270
*No*	0.78 (0.06)		1.89 (0.46)		0.70 (0.03)		1.35 (0.12)	
Systemic involvement								
*Yes*	0.78 (0.15)	0.125	1.84 (0.64) 1.63	0.198	0.64 (0.13)	0.593	1.12 (0.36)	0.755
*No*	0.73 (0.08)		(0.38)		0.62 (0.08)		1.09 (0.25)	
Hematological involvement ‡								
*Lymph node swelling*	0.80 (0.18)		1.80 (0.67)		0.62 (0.18)		1.00 (0.43)	
*Hem. Disease*	0.73 (0.06)	0.609	1.54 (0.14)	0.840	0.68 (0.02)	0.778	1.16 (0.02)	0.647
*No*	0.75 (0.11)		1.75 (0.54)		0.62 (0.09)		1.12 (0.30)	
Parotid enlargement								
*Yes*	0.83 (0.25)	0.258	1.88 (1.01)	0.613	0.61 (0.92)	0.883	1.04 (0.26)	0.727
*No*	0.75 (0.11)		1.73 (0.49)		0.62 (0.11)		1.10 (0.32)	
Glucocorticoid therapy								
*Yes*	0.71 (0.08)	0.079	1.55 (0.37) 1.70	0.133	0.61 (0.10)	0.410	1.05 (0.32)	0.384
*No*	0.76 (0.11)		(0.46)		0.64 (0.11)		1.12 (0.28)	
Disease-modifying antirheumatic drugs								
*Yes*	0.73 (0.08)	0.211	1.58 (0.36) 1.86	0.087	0.63 (0.09)	0.985	1.11 (0.31)	0.999
*No*	0.78 (0.14)		(0.63)		0.63 (0.12)		1.11 (0.32)	
Further autoimmune diseases								
*Yes*	0.71 (0.05)	0.268	1.51 (0.27)	0.221	0.54 (0.04)	0.011*	0.84 (0.12)	0.008**
*No*	0.76 (0.13)		0.18(0.58)		0.65 (0.11)		1.17 (0.32)	

^‡^ Non-normal distribution

^†^ Normal distribution. Quantitative characteristics: Spearmann’s test (non-normal distribution; rho), Pearson´s test (normal distribution; r). Qualitative characteristics: Mann–Whitney-U-test, ANOVA

^*^^−^ *** Significant difference between the two groups

*HDL* high-density lipoprotein, *LDL* low-density lipoprotein, *ENA* extractable nuclear antigens, *ESR* erythrocyte sedimentation rate, *CRP* C-reactive protein, *ANA* antinuclear antibodies, *CCA* common carotid arteries, *ICA* internal carotid artery, *RI* resistive index, *PI* pulsatility index

In the sonography subgroup, cIMT correlated strongly with age (*r* = 0.634, *p* < 0.001) and modestly with systolic blood pressure (*r* = 0.323, *p* = 0.020). cIMT was moreover higher in patients with arterial hypertension compared to patients without [1.01 ± 0.16 vs. 0.89 ± 0.17; *p* = 0.020].

Regarding Doppler indices of the common carotid arteries, CCA-RI and CCA-PI showed higher values in patients with arterial hypertension compared to patients without [0.84 ± 0.19 vs. 0.73 ± 0.06; *p* = 0.003 and 2.18 ± 0.30 vs. 1.59 ± 0.30; *p* = 0.001]. Examination of color Doppler indices of internal carotid arteries revealed significant correlations of ICA-RI and ICA-PI with age (*r* = 0.481; *p* = 0.002 and *r* = 0.457; *p* = 0.003) and systolic blood pressure (*r* = 0.465; *p* = 0.002 and *r* = 0.442; *p* = 0.004). Additionally, ICA-PI was higher in patients with arterial hypertension, compared to their hypertension-free counterparts [1.27 ± 0.27 vs. 1.05 ± 0.31; *p* = 0.004]. Interestingly, ICA-RI and ICA-PI were higher in patients with pSS and further non-rheumatological autoimmune diseases (i.e. Hashimoto’s, Basedow’s, vitiligo) [0.54 ± 0.04 vs. 0.65 ± 0.11; *p* = 0.011 and 0.84 ± 0.12 vs. 1.17 ± 0.32; *p* = 0.008].

### SCORE, cfPWV and carotid atherosclerosis

Among 119 pSS patients with a cfPWV measurement, 54 (45.38%) had cfPWV > 10 m/s, as an indicator of end-organ disease and increased aortic stiffness. 88/119 patients were eligible for the calculation of SCORE. Interestingly, out of these only 2 (2.27%) and 5 (5.68%) showed SCORE- and mSCORE-values > 5% (indicative of high risk), respectively. Of 52 patients having received a carotid US examination, 36 (69.23%) could be diagnosed with SCA. 42 of these 52 patients were also eligible for the calculation of SCORE. Similarly, out of these patients only 2 (4.76%) and 3 (7.14%) patients showed SCORE and mSCORE-values > 5%, respectively.

Notably, SCORE/mSCORE values did not show statistical differences between the patient and control group (both; *p* = 0.392).

## Discussion

### cfPWV and carotid status as markers of angiopathy and CV risk

This study revealed higher aortic stiffness and incidence of carotid atherosclerosis in patients with pSS compared to healthy controls. Importantly, these findings retained statistical significance following adjustments for confounding factors and further validation through 1:1 propensity score-matched subgroup analyses. Furthermore, our research findings indicate that mSCORE identified only a small percentage of pSS patients as being under high-risk. This underscores a significant discrepancy with the results reported in existing literature, registry data, and the gold standard marker of carotid-femoral pulse wave velocity (cfPWV), as assessed in both our study and others [5, 9]. Interestingly, cfPWV correlated in our cohort with both examined inflammation markers, and patients with concomitant non-rheumatological autoimmune conditions were found to have higher resistance and pulsatility indices of the ICA, as possible indicators of CVB risk [38].

To the best of our knowledge, this is the first study to examine the novel color Doppler indices of carotid pulsatility and resistance, next to well-established greyscale US and oscillometric markers of carotid and aortic angiopathy, in pSS. Interestingly, no significant difference was found between the groups regarding carotid compliance/stiffness, in contrast to aortic stiffness. This could be due to reduced statistical power in the subgroup analysis or the presence of atherogenic risk factors in the control group. Moreover, previous studies in patients with SLE have shown that aortic atherosclerosis and stiffness may develop earlier and to a greater extent than carotid atherosclerosis and stiffness [39], and this may also hold true for patients with pSS.

Moreover, this is the first study to show that the presence of additional non-rheumatologic autoimmune conditions can associate with higher CV/CVB risk and seemingly the largest pSS/cfPWV study to date. Interestingly, studies examining CV and CVB surrogates concomitantly with traditional CV risk scores are scarce. In particular, we are aware of only one exploration evaluating the relationship between PWV and a traditional risk calculator [Framingham Risk Score (FRS)], which however included a lower count of patients (*n* = 44) [40]. Similar to our work, statistically higher PWV values were found in patients with pSS compared to controls. FRS was however not statistically different in the two groups. In the present study, we have chosen to include cfPWV and carotid sonography indices as well as the well-established CV risk tool of SCORE, which has been suggested for CV risk evaluation by EULAR [14]. Interestingly, SCORE/mSCORE were not statistically significantly different between the two groups, pointing to a possible low predictive value of this marker in the setting of pSS.

In our exploration, not only aortic stiffness but also carotid atherosclerosis markers were statistically higher in pSS patients compared to controls. Even if cIMT has been examined in a few pSS studies, data on plaque burden are described for the first time in the present study. This is important since increased plaque burden can lead to carotid stenosis, subsequently increasing the risk for a CVB event and thus possibly worsening CV morbidity and mortality [41]. On the contrary, the predictive value of cIMT has been increasingly criticized during the last years, since increases in IMT do not seem to be an accurate marker of CVB or CV risk [42]. Despite that, the few published pSS studies on carotid atherosclerosis surrogates have focused until today on cIMT values. These works have been collectively presented by Yong et al. [5], who found that patients with pSS have overall higher IMT than healthy controls (MD = 0.08 mm; 95%CI 0.04–0.11; *p-value* < 0.01; *I*^2^ = 72%).

### Associations of CV markers among patients

In both groups, there was an association between cfPWV and higher blood pressure at the time of the measurement, which can be explained by a stretching of the artery wall due to the increased pulse pressure [43, 44]. Moreover, cfPWV and carotid sonography indices correlated strongly with age. These statistical associations are not surprising, since age is described to be an independent risk factor for CV diseases [45]. The influence of age on aortic stiffness results from structural changes in the media layer of the vessel wall during a person's lifetime. The mechanical properties of the arterial wall change with increasing age, especially due to a loss of elastic fibers and accumulation of collagen [23].

In our work, cfPWV correlated also with both examined inflammation markers (ESR and CRP), even if no statistical correlation of cfPWW with ESSDAI could be established. Interestingly, an association between aortic stiffness and acute inflammation in the context of autoimmune diseases has been extensively discussed in the literature and several potential pathophysiological mechanisms regarding the interplay between inflammation and arterial stiffening have been suggested [46]. Elevated levels of known inflammatory markers like interleukin-6 (IL-6), CRP, and interferons can directly alter the endothelial nitric oxide bioavailability [47] by impairing the vasodilatory effects of NO [48]. Moreover, these mediators can induce increased production of matrix metalloproteinases with subsequent degeneration of elastin fibers, leading to decreased arterial compliance [49]. Interestingly enough, some studies showed a reduction in cfPWV after initiation of anti-inflammatory agents [50, 51]. Furthermore, Sezis Demirci et al. were also able to show a correlation between CRP and cfPWV [52] and Novella-Navarro et al. found similarly a correlation between ESR and subclinical carotid atherosclerosis [53], highlighting the effects of inflammatory activity on the arterial tree.

Our study has some limitations. First of all, there were no longitudinal comparisons of the included CV surrogate markers with future morbidity or mortality data. Nevertheless, multiple studies were able to show that cfPWV and carotid sonography have a good CV predictive value in the general population [5, 54]. Moreover, both of these markers have been suggested for CV risk classification by the European Society of Cardiology Working Group on peripheral circulation and the ARTERY Association (level of evidence A, Recommendation IIa) [17]. Moreover, the initial patient and control cohorts were unmatched regarding some traditional CV-risk factors (with patients being older, more often females, with larger BMI, a higher prevalence of arterial hypertension, and more commonly on anti-hypertensive therapy (all; *p* ≤ 0.02)). However, to control our results, we performed propensity score matching to account for key traditional cardiovascular risk factors, including age, sex, BMI, diabetes mellitus, MAP, and smoking status. This matching confirmed a significant difference in both cfPWV and cIMT between pSS patients and controls. Moreover, our results could also be confirmed by additional regression analyses including the whole patient and control group cohort, as well as sonography subgroups, adjusting for possible CV factors that were different among patients and controls. Importantly, immunosuppressant/glucocorticoid medication in the patient group may have had a confounding effect on the findings. Even though we have thoroughly controlled the findings via statistical adjustment analyses for multiple possible confounding factors, future confirmation is warranted. A further limitation arises from the fact that sonographic examination of the carotid arteries, were included in our study design at a later point and were therefore performed in a subgroup of patients and by only one examiner. However, given that patient recruitment was consecutive and all individuals meeting the inclusion criteria were enrolled, selection bias is unlikely. The experienced examiner (serving as a certified trainer for the German Society of Ultrasound in Medicine (DEGUM)) was furthermore blinded to other surrogate measures, such as arterial stiffness, laboratory values, and the participants’ medical histories. Furthermore, the vast majority of the measured parameters (CCA-RI, CCA-PI, ICA-RI, ICA-PI) were automatically generated by the ultrasound device, minimizing the potential for examiner influence.

## Conclusions

In conclusion, this study constitutes a comprehensive exploration of surrogate CV markers in pSS, standing out as one of the most extensive investigations in this domain. It moreover represents the first examination encompassing a wide panel of angiopathy and CV risk parameters in this context. The present findings highligh the potential of vascular ultrasonographic, Doppler, and oscillometric markers to provide valuable insights into patients'arterial tree and thus assist CV risk assessment. Given their non-invasive, radiation-free, and straightforward nature, these tools may serve as valuable resources for identifying high-risk pSS patients. Nevertheless, it is crucial to emphasize the necessity for further research and longitudinal studies to achieve a more comprehensive understanding and validation of these findings.

## Supplementary information

Supplementary Material 1.

## Data Availability

No datasets were generated or analysed during the current study.

## Abbreviations

ANA: Antinuclear antibody
BMI: Body mass index
CCA: Common carotid artery
CDUS: Color Doppler
cfPWV: Carotid-femoral pulse wave velocity
cIMT: Carotid intima media thickness
CRP: C-reactive protein
CV: Cardiovascular
CVB: Cerebrovascular
EDV: End-diastolic velocity
ESR: Erythrocyte sedimentation rate
GSUS: Carotid greyscale
ICA: Internal carotid artery
MAP: Mean arterial pressure
MFV: Mean flow velocity
mSCORE: Modified SCORE
PI: Pulsatility index
pSS: Primary Sjögren´s Syndrome
PSV: Peak systolic
RA: Rheumatoid arthritis
RF: Rheumatoid factor
RI: Resistance index
RR: Relative risk
SCORE: Systematic coronary risk estimation
SICARD: Sjögren syndrome associated cardiovascular risk
SLE: Systemic lupus erythematosus
US: Ultrasound

## References

[1] Both T, Dalm VA, van Hagen PM, van Daele PL. Reviewing primary Sjogren’s syndrome: beyond the dryness - From pathophysiology to diagnosis and treatment. Int J Med Sci. 2017;14(3):191–200. 28367079 10.7150/ijms.17718 PMC5370281

[2] Kuo F, Gardener H, Dong C, Cabral D, Della-Morte D, Blanton SH, et al. Traditional cardiovascular risk factors explain the minority of the variability in carotid plaque. Stroke. 2012;43(7):1755–60. 22550054 10.1161/STROKEAHA.112.651059 PMC3383876

[3] Leone MC, Alunno A, Cafaro G, Valentini V, Marcucci E, Bartoloni E, et al. The clinical spectrum of primary Sjogren’s syndrome: beyond exocrine glands. Reumatismo. 2017;69(3):93–100. 28933131 10.4081/reumatismo.2017.1032

[4] Ramos-Casals M, Solans R, Rosas J, Camps MT, Gil A, Del Pino-Montes J, et al. Primary Sjogren syndrome in Spain: clinical and immunologic expression in 1010 patients. Medicine (Baltimore). 2008;87(4):210–9. 18626304 10.1097/MD.0b013e318181e6af

[5] Yong WC, Sanguankeo A, Upala S. Association between primary Sjogren’s syndrome, arterial stiffness, and subclinical atherosclerosis: a systematic review and meta-analysis. Clin Rheumatol. 2019;38(2):447–55. 30178172 10.1007/s10067-018-4265-1

[6] Valim V, Gerdts E, Jonsson R, Ferreira GA, Brokstad KA, Brun JG, et al. Atherosclerosis in Sjögren’s syndrome: evidence, possible mechanisms and knowledge gaps. Clin Exp Rheumatol. 2016;34(1):133–42. 26812164

[7] Cai X, Luo J, Wei T, Qin W, Wang X, Li X. Risk of Cardiovascular Involvement in Patients with Primary Sjögren’s Syndrome: a large-scale cross-sectional cohort study. Acta Reumatol Port. 2019;44(1):71–7. 31249278

[8] Bartoloni E, Baldini C, Schillaci G, Quartuccio L, Priori R, Carubbi F, et al. Cardiovascular disease risk burden in primary Sjogren’s syndrome: results of a population-based multicentre cohort study. J Intern Med. 2015;278(2):185–92. 25582881 10.1111/joim.12346

[9] Beltai A, Barnetche T, Daien C, Lukas C, Gaujoux-Viala C, Combe B, et al. Cardiovascular Morbidity and Mortality in Primary Sjogren’s Syndrome: A Systematic Review and Meta-Analysis. Arthritis Care Res (Hoboken). 2020;72(1):131–9. 30570824 10.1002/acr.23821

[10] Drosos GC, Vedder D, Houben E, Boekel L, Atzeni F, Badreh S, et al. EULAR recommendations for cardiovascular risk management in rheumatic and musculoskeletal diseases, including systemic lupus erythematosus and antiphospholipid syndrome. Ann Rheum Dis. 2022;81(6):768–79. 35110331 10.1136/annrheumdis-2021-221733

[11] Conroy RM, Pyörälä K, Fitzgerald AP, Sans S, Menotti A, De Backer G, et al. Estimation of ten-year risk of fatal cardiovascular disease in Europe: the SCORE project. Eur Heart J. 2003;24(11):987–1003. 12788299 10.1016/s0195-668x(03)00114-3

[12] Peters MJ, Symmons DP, McCarey D, Dijkmans BA, Nicola P, Kvien TK, et al. EULAR evidence-based recommendations for cardiovascular risk management in patients with rheumatoid arthritis and other forms of inflammatory arthritis. Ann Rheum Dis. 2010;69(2):325–31. 19773290 10.1136/ard.2009.113696

[13] Arts EE, Popa C, Den Broeder AA, Semb AG, Toms T, Kitas GD, et al. Performance of four current risk algorithms in predicting cardiovascular events in patients with early rheumatoid arthritis. Ann Rheum Dis. 2015;74(4):668–74. 24389293 10.1136/annrheumdis-2013-204024

[14] Agca R, Heslinga SC, Rollefstad S, Heslinga M, McInnes IB, Peters MJ, et al. EULAR recommendations for cardiovascular disease risk management in patients with rheumatoid arthritis and other forms of inflammatory joint disorders: 2015/2016 update. Ann Rheum Dis. 2017;76(1):17–28. 27697765 10.1136/annrheumdis-2016-209775

[15] Vlachopoulos C, Aznaouridis K, Stefanadis C. Aortic stiffness for cardiovascular risk prediction: just measure it, just do it! J Am Coll Cardiol. 2014;63(7):647–9. 24239659 10.1016/j.jacc.2013.10.040

[16] Triantafyllias K, Cavagna L, Klonowski A, Drott U, Fiehn C, Wendel S, et al. Possible misclassification of cardiovascular risk by SCORE in antisynthetase syndrome: results of the pilot multicenter study RI.CAR.D.A. Rheumatology (Oxford). 2021;60(3):1300–12. 32940712 10.1093/rheumatology/keaa525

[17] Vlachopoulos C, Xaplanteris P, Aboyans V, Brodmann M, Cífková R, Cosentino F, et al. The role of vascular biomarkers for primary and secondary prevention. A position paper from the European Society of Cardiology Working Group on peripheral circulation: Endorsed by the Association for Research into Arterial Structure and Physiology (ARTERY) Society. Atherosclerosis. 2015;241(2):507–32. 26117398 10.1016/j.atherosclerosis.2015.05.007

[18] Vlachopoulos C, Aznaouridis K, Stefanadis C. Prediction of cardiovascular events and all-cause mortality with arterial stiffness: a systematic review and meta-analysis. J Am Coll Cardiol. 2010;55(13):1318–27. 20338492 10.1016/j.jacc.2009.10.061

[19] Frauchiger B, Schmid HP, Roedel C, Moosmann P, Staub D. Comparison of carotid arterial resistive indices with intima-media thickness as sonographic markers of atherosclerosis. Stroke. 2001;32(4):836–41. 11283379 10.1161/01.str.32.4.836

[20] Triantafyllias K, De Blasi M, Hoffmann I, Thomaidis T, Drees P, Schwarting A. The count of tender rather than swollen joints correlates with aortic stiffness in patients with rheumatoid arthritis. Springerplus. 2016;5:428. 27104116 10.1186/s40064-016-2066-z PMC4828367

[21] Triantafyllias K, Liverakos S, Muthuraman M, Cavagna L, Parodis I, Schwarting A. Cardiovascular Risk Evaluation in Psoriatic Arthritis by Aortic Stiffness and the Systemic Coronary Risk Evaluation (SCORE): Results of the Prospective PSOCARD Cohort Study. Rheumatol Ther. 2024;11(4):897–911. 38819779 10.1007/s40744-024-00676-z PMC11265042

[22] Triantafyllias K, de Blasi M, Lütgendorf F, Cavagna L, Stortz M, Weinmann-Menke J, et al. High cardiovascular risk in mixed connective tissue disease: evaluation of macrovascular involvement and its predictors by aortic pulse wave velocity. Clin Exp Rheumatol. 2019;37(6):994–1002. 30943141

[23] Stortz M, Triantafyllias K, Schwarting A, Weinmann-Menke J. Vascular stiffness: influencing factors on carotid-femoral pulse wave velocity in systemic lupus erythematosus. Clin Exp Rheumatol. 2020;38(1):74–81. 30943131

[24] Triantafyllias K, Stortz M, de Blasi M, Leistner C, Weinmann-Menke J, Schwarting A. Increased aortic stiffness in patients with fibromyalgia: results of a prospective study on carotid-femoral pulse wave velocity. Clinical and experimental rheumatology. 2019;37 Suppl 116(1):114–5. 29185967

[25] Zardi EM, Sambataro G, Basta F, Margiotta DP, Afeltra AM. Subclinical carotid atherosclerosis in elderly patients with primary Sjögren syndrome: a duplex Doppler sonographic study. Int J Immunopathol Pharmacol. 2014;27(4):645–51. 25572746 10.1177/039463201402700422

[26] Zardi EM, Basta F, Afeltra A. Levels of Vitamin D, Disease Activity and Subclinical Atherosclerosis in Post-menopausal Women with Sjögren’s Syndrome: Does a Link Exist? In Vivo. 2016;30(5):721–5. 27566098

[27] Lee KY, Sohn YH, Baik JS, Kim GW, Kim JS. Arterial pulsatility as an index of cerebral microangiopathy in diabetes. Stroke. 2000;31(5):1111–5. 10797173 10.1161/01.str.31.5.1111

[28] Chuang SY, Cheng HM, Bai CH, Yeh WT, Chen JR, Pan WH. Blood Pressure, Carotid Flow Pulsatility, and the Risk of Stroke: A Community-Based Study. Stroke. 2016;47(9):2262–8. 27491737 10.1161/STROKEAHA.116.013207

[29] Ozisler C, Kaplanoglu H. Evaluation of subclinical atherosclerosis by ultrasound radiofrequency data technology in patients with primary Sjögren’s syndrome. Clin Rheumatol. 2019;38(3):709–17. 30334118 10.1007/s10067-018-4330-9

[30] Shiboski CH, Shiboski SC, Seror R, Criswell LA, Labetoulle M, Lietman TM, et al. 2016 American College of Rheumatology/European League Against Rheumatism Classification Criteria for Primary Sjogren’s Syndrome: A Consensus and Data-Driven Methodology Involving Three International Patient Cohorts. Arthritis rheumatology (Hoboken, NJ). 2017;69(1):35–45. 10.1002/art.39859 PMC5650478 27785888

[31] Seror R, Ravaud P, Bowman SJ, Baron G, Tzioufas A, Theander E, et al. EULAR Sjogren’s syndrome disease activity index: development of a consensus systemic disease activity index for primary Sjogren’s syndrome. Ann Rheum Dis. 2010;69(6):1103–9. 19561361 10.1136/ard.2009.110619 PMC2937022

[32] Van Bortel LM, Laurent S, Boutouyrie P, Chowienczyk P, Cruickshank JK, De Backer T, et al. Expert consensus document on the measurement of aortic stiffness in daily practice using carotid-femoral pulse wave velocity. J Hypertens. 2012;30(3):445–8. 22278144 10.1097/HJH.0b013e32834fa8b0

[33] Hickson SS, Butlin M, Broad J, Avolio AP, Wilkinson IB, McEniery CM. Validity and repeatability of the Vicorder apparatus: a comparison with the SphygmoCor device. Hypertens Res. 2009;32(12):1079–85. 19779487 10.1038/hr.2009.154

[34] Shen J, Lam SH, Shang Q, Wong CK, Li EK, Wong P, et al. Underestimation of Risk of Carotid Subclinical Atherosclerosis by Cardiovascular Risk Scores in Patients with Psoriatic Arthritis. J Rheumatol. 2018;45(2):218–26. 29142027 10.3899/jrheum.170025

[35] THOMPSON RS, TRUDINGER BJ, COOK CM. Doppler ultrasound waveform indices: A/B ratio, pulsatility index and Pourcelot ratio. BJOG: Int J Clin Obstet Gynaecol. 1988;95(6):581–8. 10.1111/j.1471-0528.1988.tb09487.x 3291936

[36] Beasley MG, Blau JN, Gosling RG. Changes in internal carotid artery flow velocities with cerebral vasodilation and constriction. Stroke. 1979;10(3):331–5. 462522 10.1161/01.str.10.3.331

[37] Ozen G, Sunbul M, Atagunduz P, Direskeneli H, Tigen K, Inanc N. The 2013 ACC/AHA 10-year atherosclerotic cardiovascular disease risk index is better than SCORE and QRisk II in rheumatoid arthritis: is it enough? Rheumatology (Oxford). 2016;55(3):513–22. 26472565 10.1093/rheumatology/kev363

[38] Hitomi Y, Masaki N, Ishinoda Y, Kagami K, Yasuda R, Toya T, et al. Effectiveness of pulsatility index of carotid Doppler ultrasonography to predict cardiovascular events. J Med Ultrason (2001). 2022;49(1):95–103. 10.1007/s10396-021-01164-5 34778938

[39] Roldan PC, Greene ER, Qualls CR, Sibbitt WL Jr, Roldan CA. Progression of atherosclerosis versus arterial stiffness with age within and between arteries in systemic lupus erythematosus. Rheumatol Int. 2019;39(6):1027–36. 30877372 10.1007/s00296-019-04267-y

[40] Sabio JM, Sánchez-Berná I, Martinez-Bordonado J, Vargas-Hitos JA, Navarrete-Navarrete N, Expósito Ruíz M, et al. Prevalence of and factors associated with increased arterial stiffness in patients with primary Sjögren’s syndrome. Arthritis Care Res (Hoboken). 2015;67(4):554–62. 25303669 10.1002/acr.22493

[41] Sillesen H, Muntendam P, Adourian A, Entrekin R, Garcia M, Falk E, et al. Carotid plaque burden as a measure of subclinical atherosclerosis: comparison with other tests for subclinical arterial disease in the High Risk Plaque BioImage study. JACC Cardiovasc Imaging. 2012;5(7):681–9. 22789936 10.1016/j.jcmg.2012.03.013

[42] Inaba Y, Chen JA, Bergmann SR. Carotid plaque, compared with carotid intima-media thickness, more accurately predicts coronary artery disease events: a meta-analysis. Atherosclerosis. 2012;220(1):128–33. 21764060 10.1016/j.atherosclerosis.2011.06.044

[43] Bruno RM, Duranti E, Ippolito C, Segnani C, Bernardini N, Di Candio G, et al. Different Impact of Essential Hypertension on Structural and Functional Age-Related Vascular Changes. Hypertension. 2017;69(1):71–8. 27802422 10.1161/HYPERTENSIONAHA.116.08041

[44] Diaz A, Tringler M, Wray S, Ramirez AJ, Cabrera Fischer EI. The effects of age on pulse wave velocity in untreated hypertension. J Clin Hypertens (Greenwich). 2018;20(2):258–65. 29267992 10.1111/jch.13167 PMC8030931

[45] Triantafyllias K, Thiele LE, Cavagna L, Baraliakos X, Bertsias G, Schwarting A. Arterial Stiffness as a Surrogate Marker of Cardiovascular Disease and Atherosclerosis in Patients with Arthritides and Connective Tissue Diseases: A Literature Review. Diagnostics (Basel). 2023;13(11). 10.3390/diagnostics13111870 PMC10252997 37296720

[46] Triantafyllias K, Thiele LE, Mandel A, Cavagna L, Baraliakos X, Bertsias G, et al. Arterial Stiffness as a Surrogate Marker of Cardiovascular Disease and Atherosclerosis in Patients with Vasculitides: A Literature Review. Diagnostics (Basel). 2023;13(24). 10.3390/diagnostics13243603 PMC10743173 38132187

[47] Yildiz M. Arterial distensibility in chronic inflammatory rheumatic disorders. Open Cardiovasc Med J. 2010;4:83–8. 20461114 10.2174/1874192401004020083 PMC2847817

[48] Jain S, Khera R, Corrales-Medina VF, Townsend RR, Chirinos JA. Inflammation and arterial stiffness in humans. Atherosclerosis. 2014;237(2):381–90. 25463062 10.1016/j.atherosclerosis.2014.09.011

[49] Arnold N, Gori T, Schnabel RB, Schulz A, Prochaska JH, Zeller T, et al. Relation between Arterial Stiffness and Markers of Inflammation and Hemostasis - Data from the Population-based Gutenberg Health Study. Sci Rep. 2017;7(1):6346. 28740206 10.1038/s41598-017-06175-2 PMC5524791

[50] Tam LS, Shang Q, Li EK, Wang S, Li RJ, Lee KL, et al. Infliximab is associated with improvement in arterial stiffness in patients with early rheumatoid arthritis – a randomized trial. J Rheumatol. 2012;39(12):2267–75. 22984272 10.3899/jrheum.120541

[51] Wong M, Oakley SP, Young L, Jiang BY, Wierzbicki A, Panayi G, et al. Infliximab improves vascular stiffness in patients with rheumatoid arthritis. Ann Rheum Dis. 2009;68(8):1277–84. 18930987 10.1136/ard.2007.086157 PMC2703705

[52] Sezis Demirci M, Karabulut G, Gungor O, Celtik A, Ok E, Kabasakal Y. Is There an Increased Arterial Stiffness in Patients with Primary Sjögren’s Syndrome? Intern Med. 2016;55(5):455–9. 26935363 10.2169/internalmedicine.55.3472

[53] Novella-Navarro M, Cabrera-Alarcón JL, Rosales-Alexander JL, González-Martín JJ, Carrión O, Peña PG. Primary Sjögren’s syndrome as independent risk factor for subclinical atherosclerosis. Eur J Rheumatol. 2022;9(1):20–5. 35110133 10.5152/eurjrheum.2021.20093 PMC10089143

[54] Spence JD, Eliasziw M, DiCicco M, Hackam DG, Galil R, Lohmann T. Carotid plaque area: a tool for targeting and evaluating vascular preventive therapy. Stroke. 2002;33(12):2916–22. 12468791 10.1161/01.str.0000042207.16156.b9

