# Supplementary material for: Oscillometric, greyscale- and novel color-Doppler-ultrasound indices of macrovascular damage in Sjögren’s: the SICARD cohort study
Source: Arthritis Res Ther. 2025 Aug 1;27:164. doi: 10.1186/s13075-025-03625-5 (PMC12317471; doi:10.1186/s13075-025-03625-5)
Supplement: Supplementary file 1 — Supplementary Material 1. [file 13075_2025_3625_MOESM1_ESM.docx]

**Table S.1.** Descriptive characteristics patient group.

|  | **n=119** |
| --- | --- |
| **ENA-Screen** *(positive)* | 73 (61.3%) |
| **SSA-Antibodies** *(positive)* | 68 (57.1%) |
| **SSB-Antibodies** *(positive)* | 19 (16.0%) |
| **Rheumatoid factor** ‡ | *positive:* 27 (22.7%)  *>triple:* 21 (17.6%) |
| **Hypergammaglobulinemia** *(positive)* | 23 (19.3%) |
| **ESR**‡ *(mm/h)* | 20 (10-38) |
| **CRP**‡ *(mg/l)* | 1.47 (0.37-4.2) |
| **ANA** ‡ *(titer)* | 320 (80-1280) |
| **Hematological involvement** ‡ | *Lymph node swelling:* 10 (8.4%)  *Haem. Disease*: 4 (3.4%) |
| **Parotid enlargement** *(yes)* | 9 (7.6%) |
| **ESSDAI** ‡ | 6 (2-15) |
| **Glucocorticoid therapy** *(yes)* | 43 (36.1%) |
| **Disease-modifying antirheumatic drugs** *(yes)*  *Monotherapy*  *Dual or more therapy*  *Hydroxychloroquine*  *Leflunomide*  *Methotrexate*  *Rituximab*  *Janus Kinase Inhibitors*  *Azathioprine*  *Belimumab*  *Methylphenolate Montefil*  *Sulfasalazine* | 53 (44.54%)  *44 (83.0%)*  *9 (17.0%)*  *33 (62.3%)*  *2 (3.8%)*  *9 (17.0%)*  *2 (3.8%)*  *1 (1.9%)*  *6 (11.3%)*  *2 (3.8%)*  *3 (5.7%)*  *1 (1.9%)* |

‡ Non-normal distribution: presentation as median (interquartile range). † Normal distribution: presentation as mean (S.D.). Others: absolute and relative frequencies.
* ^-^ *** Significant difference between the two groups.
*ENA: extractable nuclear antigens; ESR: erythrocyte sedimentation rate; CRP: C-reactive protein; ANA: antinuclear antibodies; cfPWV: Carotid-femoral pulse-wave velocity; ESSDAI: EULAR Sjögren's syndrome disease activity index.*

**Table S.2.** Results of multivariate regression analysis for carotid-femoral pulse wave velocity (cfPWV).

|  | | B | SE | Wald | df | Sig. | Exp(B) | 95% CI for Exp(B) | |
| --- | --- | --- | --- | --- | --- | --- | --- | --- | --- |
|  |  |  |  |  |  |  |  | Lower Bound | Higher Bound |
|  | cfPW | 0.329 | 0.147 | 5.046 | 1 | **0.025** | 1.390 | 1.043 | 1.852 |
|  | Age | 0.002 | 0.016 | 0.025 | 1 | 0.874 | 1.002 | 0.972 | 1.034 |
|  | Gender (1) | 1.164 | 0.573 | 4.126 | 1 | 0.042 | 3.204 | 1.042 | 9.855 |
|  | BMI | 0.065 | 0.034 | 3.669 | 1 | 0.055 | 1.067 | 0.998 | 1.140 |
|  | Hyperlipidemia(1) | -1.224 | 0.374 | 10.731 | 1 | 0.001 | 0.294 | 0.141 | 0.611 |
|  | Art. hypertension (1) | 0.011 | 0.413 | 0.001 | 1 | 0.978 | 1.011 | 0.450 | 2.274 |
|  | Diabetes mellitus(1) | -0.889 | 1.254 | 0.502 | 1 | 0.479 | 0.411 | 0.035 | 4.801 |
|  | Smoking (1) | -20.817 | 11323.158 | 0.000 | 1 | 0.999 | 0.000 | 0.000 |  |
|  | Smoking (2) | -21.041 | 11323.158 | 0.000 | 1 | 0.999 | 0.000 | 0.000 |  |
|  | Constant | 17.482 | 11323.158 | 0.000 | 1 | 0.999 | 39124797.300 |  |  |

***Table S.3.*** Results of a statistical adjustment model for carotid-femoral pulse wave velocity (cfPWV).

|  | B | SE | T statistic | Sig. | 95% CI for B | |
| --- | --- | --- | --- | --- | --- | --- |
|  |  |  |  |  | Lower Bound | Upper Bound |
| (Intercept) | 7.996 | 1.319 | 6.060 | 0.000 | 5.369 | 10.622 |
| Patient | 0.687 | 0.343 | 1.999 | **0.049** | 0.003 | 1.370 |
| Age (≥ 55) | 1.629 | 0.395 | 4.129 | 0.000 | 0.844 | 2.414 |
| Gender | 0.334 | 0.543 | 0.614 | 0.541 | -0.747 | 1.415 |
| BMI (≥ 35) | 0.130 | 0.836 | 0.156 | 0.876 | -1.533 | 1.794 |
| Art. hypertension | 0.729 | 0.715 | 1.019 | 0.311 | -0.695 | 2.152 |
| Antihypertensive therapy | 0.493 | 0.700 | 0.704 | 0.483 | -0.900 | 1.885 |
| Heart rate | -0.010 | 0.016 | -0.594 | 0.554 | -0.042 | 0.023 |

***Table S.4***. Descriptive characteristics patients (carotid sonography subgroups).

|  | **n=52** |
| --- | --- |
| **ENA-Screen** *(positive)* | 35 (67.3%) |
| **SSA-Antibodies** *(positive)* | 34 (65.4%) |
| **SSB-Antibodies** *(positive)* | 12 (23.1%) |
| **Rheumatoid factor** ‡ | *positive:* 17 (32.7%)  *>triple:* 9 (17.3%) |
| **Hypergammaglobulinemia** *(positive)* | 7 (13.5%) |
| **ESR**‡ *(mm/h)* | 14 (9.25-39.5) |
| **CRP**‡ *(mg/l)* | 1.2 (0.22-2.79) |
| **ANA** ‡ *(titer)* | 320 (80-1280) |
|  |  |
| **Focus Score** *(1/4mm^2^)* | 2.65 (1.8-3.56) |
| **Sicca symptoms** *(yes)* | 47 (90.4%) |
| **Saxon´s test** *(positive)* | 10 (19.2%) |
| **Saxon´s test difference**‡ *(g)* | 2.40±1.67 |
| **Schirmer´s test** *(positive)* | 23 (44.2%) |
| **Schirmer´s test** (lowest value *(mm)*) ‡ | 6.20±8.53 |
| **Systemic involvement** *(yes)* | 25 (48.1%) |
| **Heamatological involvement** ‡ | *Lymph node swelling:*  8 (15.4%)  *Haem. disease*: 2 (3.8%) |
| **Parotid enlargement** *(yes)* | 4 (7.7%) |
| **ESSDAI** ‡ | 6 (0-17.75) |
| **Glucocorticoid therapy** *(yes)* | 16 (30.8%) |
| **Disease-modifying antirheumatic drugs** *(yes)* | 24 (46.15%) |

‡ Non-normal distribution: presentation as median (interquartile range). † Normal distribution: presentation as mean (S.D.). Others: absolute and relative frequencies.
* ^-^ *** Significant difference between the two groups.
*ENA: extractable nuclear antigens; ESR: erythrocyte sedimentation rate; CRP: C-reactive protein; ANA: antinuclear antibodies; ESSDAI: EULAR Sjögren's syndrome disease activity index; cIMT: carotid intima-media thickness.*

**Table S.5.** Results of multivariate regression analysis for carotid intima-media thickness (cIMT).

|  | | B | SE | Wald | df | Sig. | Exp(B) | 95% CI for Exp(B) | |
| --- | --- | --- | --- | --- | --- | --- | --- | --- | --- |
|  |  |  |  |  |  |  |  | Lower Bound | Higher bound |
|  | cIMT | 2.329 | 0.623 | 13.985 | 1 | **<0.001** | 10.27 | 3.03 | 34.81 |
|  | Age | -0.111 | 0.04 | 7.712 | 1 | 0.005 | 0.895 | 0.828 | 0.968 |
|  | Gender (1) | 2.384 | 0.996 | 5.733 | 1 | 0.017 | 10.848 | 1.541 | 76.366 |
|  | Hyperlipidemia (1) | -2.432 | 0.77 | 9.983 | 1 | 0.002 | 0.088 | 0.019 | 0.397 |
|  | Art. hypertension (1) | 0.912 | 0.685 | 1.772 | 1 | 0.183 | 2.489 | 0.65 | 9.531 |
|  | Smoking (1) | -20.287 | 20265.007 | 0 | 1 | 0.999 | 0 | 0 |  |
|  | Smoking (2) | -20.435 | 20265.007 | 0 | 1 | 0.999 | 0 | 0 |  |
|  | Diabetes Mellitus (1) | 0.13 | 1.284 | 0.01 | 1 | 0.919 | 1.139 | 0.092 | 14.115 |
|  | Constant | 25.448 | 20265.007 | 0 | 1 | 0.999 | 1.13E+11 |  |  |

***Table S.6.*** Results of a statistical adjustment model for carotid-Intima-Media Thickness (cIMT).

|  | estimate | SE | T statistic | Sig. | 95% CI for B | |
| --- | --- | --- | --- | --- | --- | --- |
|  |  |  |  |  | Lower Bound | Upper Bound |
| (Intercept) | 0.446 | 0.210 | 2.128 | 0.039 | 0.024 | 0.868 |
| Patient | 0.134 | 0.040 | 3.346 | **0.002** | 0.053 | 0.215 |
| Gender | 0.144 | 0.063 | 2.276 | 0.028 | 0.017 | 0.271 |
| Mean arterial pressure | 2.187 | 4.422 | 0.495 | 0.623 | -6.714 | 11.089 |
| Diastolic arterial pressure | -1.462 | 2.948 | -0.496 | 0.622 | -7.397 | 4.473 |
| Systolic arterial pressure | -0.725 | 1.474 | -0.492 | 0.625 | -3.692 | 2.242 |
